# Bulky 2,6-disubstituted aryl siloxanes and a disilanamine

**DOI:** 10.1107/S2056989020001413

**Published:** 2020-02-06

**Authors:** Flavia Marszaukowski, Karen Wohnrath, René T. Boeré

**Affiliations:** a Departamento de Química, Universidade Estadual de Ponta Grossa, 84030-900, Ponta Grossa, Paraná, Brazil; b Chemistry and Biochemistry, University of Lethbridge, 4401 University Drive West, Lethbridge, Alberta, T1K 3M4, Canada

**Keywords:** crystal structure, steric pressure, *ortho*-disubstitution, out of plane distortion, high symmetry

## Abstract

The crystal structures of 5-bromo-1,3-di-*tert-*butyl-2-[(tri­methyl­sil­yl)­oxy]benzene, C_17_H_29_BrOSi, (I), 1,3-di-*tert-*butyl-2-[(tri­methyl­sil­yl)­oxy]benzene, C_17_H_30_OSi, (II), and *N*-(2,6-diiso­propyl­phen­yl)-1,1,1-trimethyl-*N*-(tri­methyl­sil­yl)silanamine, C_18_H_35_NSi_2_, (III), are reported. Compound (I) crystallizes in space group *P*2_1_/*c* with *Z*′ = 1, (II) in *Pnma* with *Z*′ = 0.5 and (III) in *Cmcm* with *Z*′ = 0.25. Consequently, the mol­ecules of (II) are constrained by *m* and those of (III) by *m*2*m* site symmetries. Despite this, both (I) and (II) are distorted towards mild boat conformations, as is typical of 2,6-di-*tert-*butyl-substituted phenyl compounds, reflecting the high local steric pressure of the flanking alkyl groups. Compound (III) by contrast is planar and symmetric, and this lack of distortion is compatible with the lower steric pressure of the flanking 2,6-diisopropyl substituents.

## Abstract 2

High steric constraints lead to inter­locking tri­methyl­silyl and tertbutyl (or isoprop­yl) substituents in the structures of three silyl-protected phenol and anilene reagents. The two di-*tert*-butyl compounds are distorted from planarity to mild boat conformations but the diiso­propyl­anilene remains rigorously planar.

## Chemical context   

Aryl siloxanes and silanamines are important reaction inter­mediates, especially as protecting groups for phenols and anilines (Lucente-Schultz *et al.*, 2009 ▸). Thus, 5-bromo-1,3-di-*tert-*butyl-2-[(tri­methyl­sil­yl)­oxy]benzene, (I), is used as a synthetic inter­mediate to form *inter alia p*-conjugated aryl­boron radicals (Chung *et al.*, 2018 ▸), *p*-quinone methides (Wang *et al.*, 2018 ▸) and spin-labelled polymers (Otaki & Goto, 2019 ▸). Recently a new cross-coupling reaction using the parent phenol was shown to be more effective than using protected 1,3-di-*tert-*butyl-2-[(tri­methyl­sil­yl)­oxy]benzene, (II) (Nieves-Quinones *et al.*, 2019 ▸). Silanamines such as *N*-(2,6-diiso­propyl­phen­yl)-1,1,1-trimethyl-*N*-(tri­methyl­sil­yl)silanamine, (III), can support chemistry at the 4-position of the ring, including robust heteroelement derivatives (Maaninen *et al.*, 1999 ▸), and are also good sources for the controlled synthesis of early transition-metal amides (Siemeling *et al.*, 1999 ▸; Pennington *et al.*, 2005 ▸). Similarly, substitution of (II) at the 4-position of the ring leads to numerous intrinsic heteroatom derivatives in addition to follow-up reactivity at oxygen (Kindra *et al.*, 2013 ▸; Poverenov *et al.*, 2007 ▸; Satoh & Shi, 1994 ▸; Healy & Barron, 1990 ▸). Herein we report the single-crystal X-ray diffraction structures of (I), (II) and (III).

## Structural commentary   

**Figure 1:**
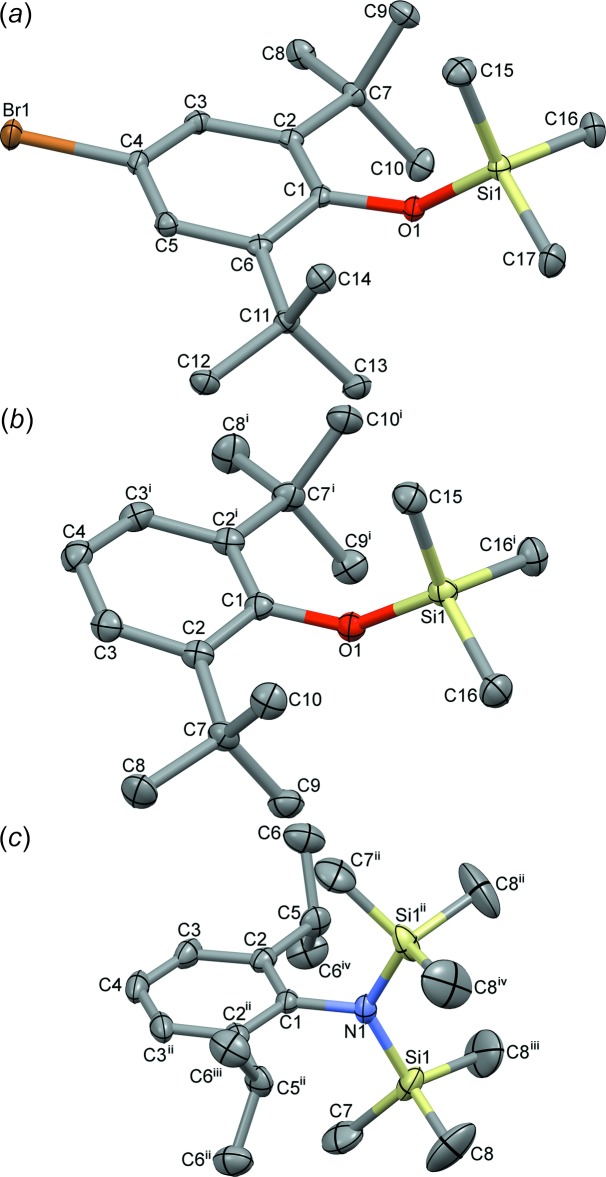
Displacement ellipsoids plot of the mol­ecular structures of (*a*) (I) at the 50% probability level; (*b*) (II), also at the 50% probability level, and (*c*) (III) at the 40% probability level. H atoms have been omitted and the atom numbering schemes are shown. [Symmetry codes: (i): *x*, 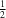 − *y*, *z*; (ii) 1 − *x*, *y*, 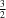 − *z*; (iii) *x*, *y*, 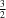 − *z*; (iv) 1 − *x*, *y*, *z*.]

Compound (I) crystallizes on a general position in *P*2_1_/*c* and adopts a distortion towards boat-shaped (Fig. 1 ▸ *a*) in which all of the atoms along the central ridge of the substituted benzene ring tilt above a best plane defined by the central C2/C3/C5/C6 ring carbon atoms, whilst the ^*t*^Bu groups tilt below. Inter­estingly, (II) crystallizes with a similar degree of distortion towards a boat conformation (Fig. 1 ▸ *b*): deviations from the central planes in (I) and (II) are 1.401 (6) and 1.446 (5) Å for Si1, 0.226 (4) and 0.227 (3) Å for O1, 0.111 (3) and 0.107 (2) Å for C1, 0.039 (3) and 0.040 (3) Å for C4 and an average of −0.117 (4) and −0.112 (2) Å for the two ^*t*^Bu central carbon atoms; note, however, that (II) has bilateral symmetry from the occupation of Wyckoff site 4*c* in *Pnma* with Si1, O1, C1, C4 and C15 on the mirror. The Si1—O1 bond lengths in (I) and (II) are closely comparable at 1.6617 (15) and 1.6655 (12) Å, respectively, as are the C1—O1 lengths at 1.379 (2) and 1.3821 (19) Å. Noticeably, all these dimensions are long, corresponding to the upper quartiles of the compiled values (1.652 and 1.373 Å, respectively; Lide, 2004 ▸) for all organic Si—O bond lengths. In both mol­ecules, the Me_3_SiO groups are strongly tilted out of the mol­ecular planes and the C1—O1—Si1 angles are similar but not identical at 139.75 (13) and 137.9 (1)°. Consideration of space-filling representations strongly suggest that these angles allow the best fitting of the bulky Me_3_Si groups between flanking ^*t*^Bu groups, with very specific orientations of the H atoms on all the components.

In contrast to the two siloxanes, the silanamine (III) is rigorously planar with the N(SiMe_3_)_2_ moiety strictly orthogonal to the aryl ring (Fig. 1 ▸ *c*) as required by *m*2*m* symmetry at Wyckoff site 4*c* in space group *Cmcm*. Consideration of a space-filling model also confirms the tight fit of the two Me_3_Si groups between the flanking isopropyl moieties, and the constraints on orientations of the Me groups of all the substituents are also considerable, inducing a constrained inter­nal orientation in (III). The N1—Si1 bond lengths are 1.7529 (13) Å, approaching the upper quartile of the compiled standard values of 1.755 Å for all aromatic N—Si bond lengths (Lide, 2004 ▸). The C1—N1—Si1 angles are 116.92 (7)°, considerably smaller than the C—O—Si angles in (I) and (II), consistent with trigonal substitution at N1.
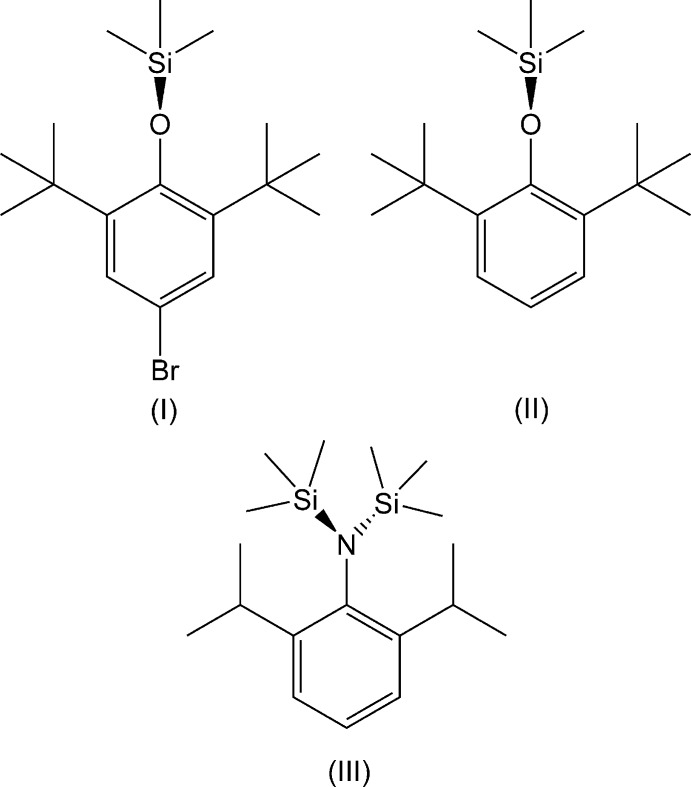


The close inter­locking of the methyl group atoms belonging to the ^*t*^Bu/^*i*^Pr and Me_3_Si substituents in all three mol­ecules is very evident in Fig. 1 ▸.

## Supra­molecular features   

**Figure 2:**
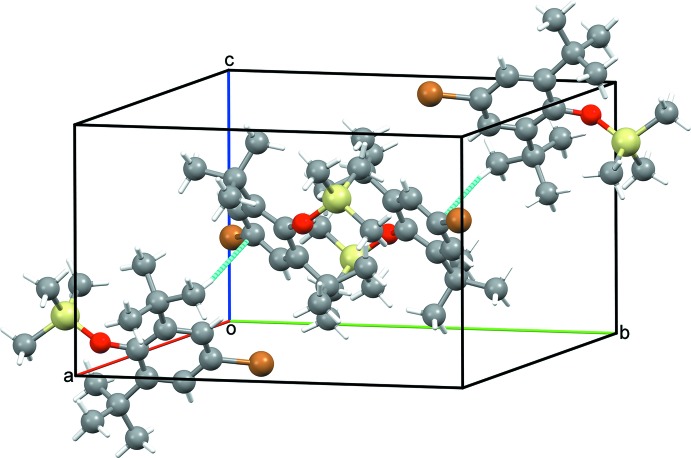
Unit-cell packing diagram for (I) viewed bisecting γ with H atoms shown with arbitrary radii and intermolecular contacts less than ΣrvdW as dashed blue lines.

**Figure 3:**
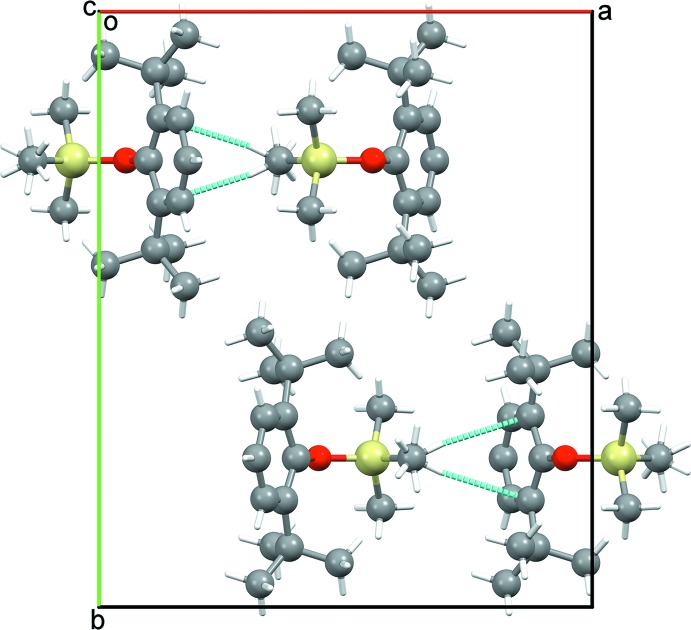
Unit-cell packing diagram for (II) viewed perpendicular to *c* with H atoms shown with arbitrary radii and intermolecular contacts less than ΣrvdW as dashed blue lines.

**Figure 4:**
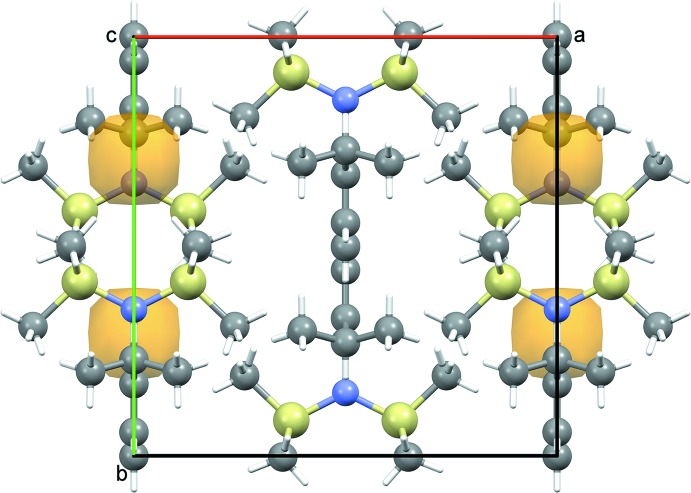
Unit-cell packing diagram for (III) viewed perpendicular to *c* with H atoms shown with arbitrary radii. Small, non solvent-accessible, voids of 22 Å^3^ are shaded ochre.

Compound (I) is gently packed (Fig. 2 ▸) in its extended structure with few contacts shorter than Σ*r*_vdW_. By contrast, (II) forms stacks along the *a-*axis direction (Fig. 3 ▸) with some contacts from SiMe_3_ H atoms to aromatic rings at 2.80 Å, within (Σ*r*_vdW_ – 0.1 Å), indicative of weak dispersion inter­actions; this is consistent with the high crystallinity encountered when (II) is a synthetic by-product. The structure of (III) has very high symmetry as a consequence of space group *Cmcm* and Z′ = 0.25 but there are no contacts shorter than Σ*r*_vdW_. The resultant weak packing (Fig. 4 ▸) may be a contrib­uting factor to the rather large displacement ellipsoids occurring in the anisotropic refinement of (III).

## Database survey   

The geometry of (I) may be compared to that of 4-bromo-2,6-di-*tert*butyl­phenol, for which a modern low-temperature area-detector structure has been reported in the Cambridge Structure Database (CSD, Version 5.40, with updates to February 2019; Groom *et al.*, 2016 ▸) with refcode BBPHOL02 (Marszaukowski & Boeré, 2019 ▸). The C—Br distance of 1.904 (2) Å in (I) is indistinguishable from 1.905 (3) Å in the latter at the 99% confidence level. Both (I) and (II) can be compared with five other reported structures in the CSD that share the same combination of 2,6-di-*tert-*butyl­phenyl rings and 1-tri­methyl­siloxane substituents, with CSD refcodes: GIFCEE (Poverenov *et al.*, 2007 ▸), JEHDOP (Healy & Barron, 1990 ▸), LIKYEJ, which has three independent such moieties attached to a B_3_O_3_ ring (Satoh & Shi, 1994 ▸), TIXZUK, in which two such groups are attached to bis­muth atoms that are dimerized through a short *M*⋯*M* contact (Kindra *et al.*, 2013 ▸) and TIYBEK (Kindra *et al.*, 2013 ▸). All the inter­atomic distances and angles in (I) and in (II) are indistinguishable from the mean values for the eight independent comparators at the 99% confidence level (nine when BBPHOL02 is included for the non-tri­methyl­silyl dimensions). This allows for the computation of global mean values (Table 1 ▸). Thus, the Si—O distances of 1.6617 (15) and 1.6655 (12) Å in (I) and (II) fit within an average of 1.657 (10) Å for this set of di-^*t*^Bu-flanked tri­methyl­siloxanes, and close to the upper quartile value of 1.652 Å for all organic Si—O bond lengths (Lide, 2004 ▸). A comparison of symmetry-averaged inter­atomic distances (Å) and angles (°) for (I), (II) and (III) with the discussed comparator sets is presented in Table 1 ▸.

One exception to taking meaningful averages concerns the C1—O1—Si1 angles, which though similar in (I) and (II) at 139.75 (13) and 137.91 (10)°, are both inter­mediate with respect to an overall range from a low of 126.8 (1) in GIFCEE to a high of 150.3 (2)° in one of the TIKZUK components. Evidently, this angle has a wide variability and a low specificity, so it was of inter­est to investigate if the values are independent of other structural parameters. For example, attempted correlation of these angles with the C1—O1 bond length shows an almost random scatter. However, *all* members of this series show mild distortions of the substituted benzene rings towards a *boat* conformation in which S11, O1, C1 and C4 deviate in the same direction from planar and the ^*t*^Bu group C7 and C11 atoms deviate in the opposite direction. A strong correlation is found between the deviation of Si1 from the mean planes defined by C2, C3, C5 and C6 (and hence with the C1—O1—Si1 angle) and similar deviations of smaller magnitude for O1, C1 and C4 (correlation coefficients of 0.98, 0.93 and 0.83, respectively). Thus, bends at the siloxane oxygen atoms smoothly pucker the whole rings toward boat conformations. A consideration of the fits between the ^*t*^Bu and Me_3_Si groups also indicates that the former undergo rotation so as to accommodate the various tilt angles of the latter from the mean mol­ecular planes – a double turnstile motion that accommodates variations in their relative positions despite the inter­locking inter­actions within these structures.

A close structural analogue to (III) has been reported for an amino­silanetri­thiol analogue (IV[Chem scheme2]), which has one of the SiMe_3_ groups replaced by Si(SH)_3_) (CSD refcode QOCSEI; Li *et al.*, 2014 ▸). This is almost isostructural and crystallizes in space group *Cmc*2_1_ with a unit cell that is imperceptibly different at the 99% confidence level (0.6% shorter in *a* but 0.6% longer in *c*, leading to a volume just 0.1% lower). It has a mirror disorder of the SiMe_3_ and Si(SH)_3_ groups as a consequence of being positioned with the aryl ring on a lattice mirror plane. Mol­ecules of (IV) share the same relative lattice positions as those of (III) in *Cmcm*.
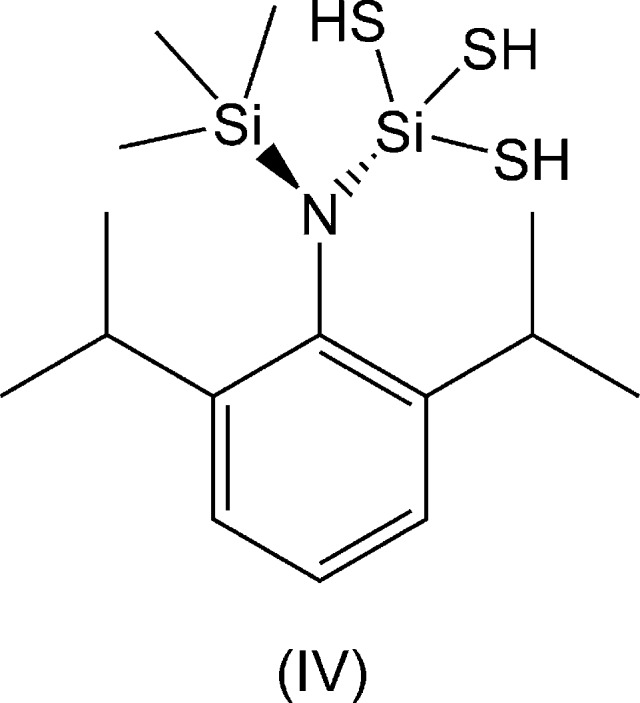


The reduced site symmetry [compared to *m*2*m* in (III)] results in considerable asymmetry in the benzene ring in QOCSEI and a small deviation from full orthogonality of the N-Si*R*_3_ units w.r.t. the benzene ring (dihedral angle of 88.1°). By contrast, orthogonal arrangements of the aryl and CNSi_2_ planes are found in the (ordered) structures of two ring-substituted derivatives of (III) with refcodes CORKAV (4-SeCl_3_) and QOCSEI (4-ferro­cen­yl­ethyn­yl), neither of which have site-symmetry restraints (Maaninen *et al.* 1999 ▸; Siemeling *et al.*, 1999 ▸). This suggests that it is the inter­locking steric constraints of the 2,6-diisopropyl and N(SiMe_3_)_2_ groups that induces these highly regular structures, and greater planarity of the aromatic rings and substituents compared to the typical distortions observed for 2,6-di-*tert-*butyl phenol derivatives such as (I) and (II). Notably, there is only one reported crystal structure of a 2,6-di-*tert*-butyl­aniline with two silyl substituents, in the form of a four-membered N_2_(Si^*i*^Pr_2_)_2_ ring (refcode: FOTWEQ; Stalke *et al.*, 1987 ▸) and this is severely distorted from planarity towards a boat conformation with the N atoms 0.60 and 0.69 Å out of the planes of the four central ring carbon atoms.

Within the comparison set of these three previously reported structures, the Si1—N1 distance of 1.7529 (13) Å in (III) compares well with the mean value of 1.750 (2) Å for CAQWUW and CORKAV, whereas the value in QOCSEI of 1.788 (8) Å is different at the 99% confidence level and may have been elongated by the disorder refinement, in agreement with the author’s report that the DFT-computed value for this bond is noticeably shorter at 1.744 Å (Li *et al.*, 2014 ▸). There is also considerable variation amongst the four structures for the individual Si—C lengths in different positions (*e.g.* Si1—C7 *versus* Si1—C8) but the *average* of Si—C distances of 1.861 (2) Å, and N—Si—C and C—Si—C angles of 110.76 (11) and 107.79 (13)°, respectively in (III) are not significantly different at the 99% confidence level from the corresponding mean values for the comparison set of 1.859 (6) Å, 110.7 (5) and 107.84 (9)°, respectively. All other inter­atomic distances and angles found for (III) are similarly indistinguishable from the comparison set at the 99% confidence level.

## Synthesis and crystallization   

2,6-Di-*tert*-butyl-phenol (Acros), 2,6-di-*tert*-butyl-4-bromo­phenol and 2,6-diiso­propyl­anilene (Aldrich) were commercial products and used as received except where noted. The technical grade anilene was purified by vacuum distillation. Solvents (BDH) were chromatographic grade and dried before use by standard methods. NMR spectra were recorded on a 300 MHz Bruker Avance II spectrometer and are referenced to tetra­methyl­silane at 0 (^1^H) and CDCl_3_ at 77.23 (^13^C) ppm.

### Preparation of (I)   

Compound (I) was prepared by modification of a literature method (Lucente-Schultz *et al.*, 2009 ▸). A 250 ml side-arm RBF was charged with 2.85 g (10 mmol) of 2,6-di-*tert*-butyl-4-bromo­phenol in 50 ml of dry THF. The solution was cooled to 195 K for 10 min with stirring. Then 6.0 ml (15 mmol) ^*n*^BuLi (2.5 *M* in hexa­nes) was slowly added and the resulting mixture was stirred for 1 h. Next, chloro­trimethilsylane (2.17 g, 20 mmol) was added to the mixture and the reaction was stirred for 1 h while warming to RT. The product was poured into water (50 ml) and extracted with hexa­nes twice (2 × 20 ml). The organic layer was washed with water (30 ml), dried with anhydrous MgSO_4_ and filtered. The product was isolated as a colorless crystalline solid on evaporation and found to be synthetically pure. Yield 3.21 g (90%). ^1^H NMR (300.13 MHz, CDCl_3_): δ 0.41 (SiC*H*_3_, *s*, 9H); 1.38 [C(C*H*_3_)_3_, *s*, 18H]; 7.32 (C*H*, *s*, 2H). ^13^C NMR (75.48 MHz, CDCl_3_): δ 3.90 (Si*C*H_3_); 31.04 [C(*C*H_3_)_3]_; 35.31 [–*C*(CH_3_)_3_]; 113.82 (C_4_); 128.69 (C_3,5_); 143.12 (C_2_,_6_); 152.44 (C_1_). Crystals were grown from hexa­nes.

### Preparation of (II)   

Compound (II) was prepared in an analogous manner to (I) from 2.06 g (10 mmol) of 2,6-di-*tert*-butyl­phenol. Other reagent qu­anti­ties match those used for (I). The colorless crystalline product solidified on evaporation and was found to be synthetically pure (1.81 g, 65%). ^1^H NMR (300.13 MHz, CDCl_3_): δ 0.41 (SiC*H*_3_, *s*, 9H); 1.41 [C(C*H*_3_)_3_, *s*, 18H]; 6.86 (C*H*, *t*, 1H, *J*_H–H_ = 7.9 Hz); 7.25 (C*H*, *d*, 2H, *J*_H–H_ = 7.8 Hz). ^13^C NMR (75.48 MHz, CDCl_3_): δ 4.03 (Si*C*H_3_); 31.33 (C(*C*H_3_)_3_); 35.18 [–*C*(CH_3_)_3_]; 120.69 (C_3_); 125.80 (C_4_); 140.87 (C_2_); 153.20 (C_1_). NMR data was compared to the literature values (Goyal & Singh, 1996 ▸). Crystals were grown from hexa­nes.

### Preparation of (III)   

Compound (III) was prepared as reported in the literature (Maaninen *et al.*, 1999 ▸). Crystals were grown by sublimation. ^1^H NMR agrees with the literature.

## Refinement   

Crystal data, data collection and structure refinement details are summarized in Table 2 ▸. All H atoms in the three structures are attached to C atoms and are treated as riding, with C—H = 0.98 Å and *U*_iso_ = 1.5*U*_eq_(C) for methyl, with C—H = 0.97 Å and *U*_iso_ = 1.3*U*_eq_(C) for methine and with C—H = 0.95 Å and *U*_iso_ = 1.2*U*_eq_(C) for aromatic. H atoms attached to methyl carbon atoms C15 in the structure of (II) and C7 in the structure of (III) are duplicated by the mirror symmetries and have been refined with half-occupancy.

## Supplementary Material

Crystal structure: contains datablock(s) I, II, III. DOI: 10.1107/S2056989020001413/hb7886sup1.cif

Structure factors: contains datablock(s) I. DOI: 10.1107/S2056989020001413/hb7886Isup2.hkl

Structure factors: contains datablock(s) II. DOI: 10.1107/S2056989020001413/hb7886IIsup3.hkl

Structure factors: contains datablock(s) III. DOI: 10.1107/S2056989020001413/hb7886IIIsup4.hkl

Supporting information file. DOI: 10.1107/S2056989020001413/hb7886IIIsup5.mol

Supporting information file. DOI: 10.1107/S2056989020001413/hb7886Isup6.cml

Supporting information file. DOI: 10.1107/S2056989020001413/hb7886IIsup7.cml

Supporting information file. DOI: 10.1107/S2056989020001413/hb7886IIIsup8.cml

CCDC references: 1981235, 1981234, 1981233

Additional supporting information:  crystallographic information; 3D view; checkCIF report

## Figures and Tables

**Table 1:** Average inter­atomic distances and angles (Å, °) in (I), (II) and (III) with comparators Atom numbers taken from (I).

Parameter	(I)	(II)	Mean siloxane^*a*^	(III)	Mean silanamine^*b*^	
Si1—O1,N1	1.6617 (15)		1.657 (10)	1.7529 (13)	1.762 (18)	
Ave Si—C	1.865 (2)	1.8666 (15)	1.861 (8)	1.861 (2)	1.859 (6)	
C1—O1,N1	1.379 (2)	1.3823 (19)	1.385 (7)	1.448 (4)	1.453 (8)	
Av C1—C2,6	1.419 (3)	1.4183 (14)	1.415 (5)	1.405 (3)	1.403 (4)	
Av C2,5—C3,6	1.395 (3)	1.3977 (17)	1.395 (7)	1.398 (3)	1.389 (3)	
Av C2,6—C7,11	1.542 (3)	1.5459 (16)	1.546 (4)	1.513 (3)	1.519 (7)	
Av C3,5—C4	1.379 (3)	1.3798 (15)	1.385 (6)	1.373 (3)	1.378 (11)	
Av C7,11-meth­yl	1.540 (3)	1.5386 (17)	1.537 (7)	1.530 (2)	1.523 (6)	
						
Av C—Si—C	109.88 (9)	109.21 (6)	110.0 (8)	110.76 (11)	110.7 (5)	
Av O,*N*—Si—C	109.01 (11)	109.68 (7)	108.9 (18)	107.79 (13)	107.84 (9)	
C1—O1,N1—Si1	139.75 (13)	137.90 (10)	140 (5)	116.92 (7)	117.0 (10)	
Av C2,6—C1—O,*N*	119.42 (17)	119.31 (7)	119.2 (10)	119.81 (13)	119.8 (2)	
C2—C1—C6	120.97 (18)	121.23 (15)	121.4 (5)	120.4 (3)	120.5 (4)	
Av C1—C2,6—C3,5	117.75 (18)	117.12 (11)	117.9 (6)	118.5 (2)	118.7 (4)	
Av C1—C2,6—C7,11	123.92 (17)	124.64 (11)	124.3 (10)	123.05 (19)	123.2 (4)	
Av C3,5—C2,6—C7,C11	118.34 (17)	118.24 (10)	112.5 (13)	118.5 (2)	118.6 (11)	
Av C2,6—C3,5—C4	120.59 (18)	122.25 (12)	122.5 (11)	121.6 (2)	121.1 (10)	
C3—C4—C5	121.40 (18)	119.17 (16)	118.5 (16)	119.5 (3)	119.9 (16)	
Av C2,6—C7,11—Me	110.79 (16)	110.86 (10)	110.7 (11)	111.78 (14)	111.83 (15)	
Av Me—C7,11—Me	108.12 (16)	108.04 (10)	108.2 (14)	109.5 (2)	109.55 (7)	

Notes: (*a*) Mean values taken over (I), (II), BBPHOL02, GIFCEE, JEHDOP, LIKYEJ, TIXZUK and TIYBEK, treating crystallographically independent entities separately. (*b*) Mean values taken over (III), CAQWUW, CORKAV and QOCSEI.

**Table 2:** Experimental details

	(I)	(II)	(III)
Crystal data			
Chemical formula	C_17_H_29_BrOSi	C_17_H_30_OSi	C_18_H_35_NSi_2_
*M*_r_	357.40	278.50	321.65
Crystal system, space group	Monoclinic, *P*2_1_/*c*	Orthorhombic, *P* *n* *m* *a*	Orthorhombic, *C* *m* *c* *m*
Temperature (K)	100	100	173
*a*, *b*, *c* (Å)	13.0955 (1), 15.3457 (2), 9.0449 (1)	14.47237 (14), 17.4657 (2), 6.73933 (7)	12.199 (3), 12.091 (3), 14.177 (3)
α, β, γ (°)	90, 94.617 (1), 90	90, 90, 90	90, 90, 90
*V* (Å^3^)	1811.76 (3)	1703.50 (3)	2091.1 (8)
*Z*	4	4	4
Radiation type	Cu *K*α	Cu *K*α	Mo *K*α
μ (mm^−1^)	3.67	1.13	0.17
Crystal size (mm)	0.42 × 0.21 × 0.13	0.31 × 0.11 × 0.07	0.19 × 0.16 × 0.10

Data collection			
Diffractometer	Rigaku Oxford Diffraction SuperNova, Dual, Cu at zero, Pilatus 200K	Rigaku Oxford Diffraction SuperNova, Dual, Cu at zero, Pilatus 200K	Bruker APEXII CCD
Absorption correction	Multi-scan (*CrysAlis PRO*; Rigaku OD, 2018 ▸)	Multi-scan (*CrysAlis PRO*; Rigaku OD, 2018 ▸)	ψ scan (*SADABS*; Bruker, 2014 ▸)
*T*_min_, *T*_max_	0.507, 1.000	0.773, 1.000	0.667, 0.746
No. of measured, independent and observed [*I* > 2σ(*I*)] reflections	20327, 3935, 3924	17855, 1793, 1663	8996, 1317, 1082
*R*_int_	0.024	0.046	0.039
(sin θ/λ)_max_ (Å^−1^)	0.639	0.626	0.652

Refinement			
*R*[*F* ^2^ > 2σ(*F* ^2^)], *wR*(*F* ^2^), *S*	0.031, 0.073, 1.10	0.033, 0.093, 1.06	0.047, 0.126, 1.04
No. of reflections	3935	1793	1317
No. of parameters	191	99	64
No. of restraints	0	0	54
H-atom treatment	H-atom parameters constrained	H-atom parameters constrained	H-atom parameters constrained
Δρ_max_, Δρ_min_ (e Å^−3^)	0.66, −0.37	0.26, −0.28	0.36, −0.26

Computer programs: *CrysAlis PRO* (Rigaku OD, 2018 ▸), *APEX2* and *SAINT* (Bruker, 2014 ▸), *SHELXT* (Sheldrick, 2015*a* ▸), *SHELXL* (Sheldrick, 2015*b* ▸), *OLEX2* (Dolomanov *et al.*, 2009 ▸) and *publCIF* (Westrip, 2010 ▸).

## supplementary crystallographic information

### 5-Bromo-1,3-di-tert-butyl-2-[(trimethylsilyl)oxy]benzene (I) Crystal data

**Table:**

C_17_H_29_BrOSi	*F*(000) = 752
*M**_r_* = 357.40	*D*_x_ = 1.310 Mg m^−^^3^
Monoclinic, *P*2_1_/*c*	Cu *K*α radiation, λ = 1.54184 Å
*a* = 13.0955 (1) Å	Cell parameters from 18499 reflections
*b* = 15.3457 (2) Å	θ = 4.4–79.8°
*c* = 9.0449 (1) Å	µ = 3.67 mm^−^^1^
β = 94.617 (1)°	*T* = 100 K
*V* = 1811.76 (3) Å^3^	Prism, clear colourless
*Z* = 4	0.42 × 0.21 × 0.13 mm

### 5-Bromo-1,3-di-tert-butyl-2-[(trimethylsilyl)oxy]benzene (I) Data collection

**Table:**

Rigaku Oxford Diffraction SuperNova, Dual, Cu at zero, Pilatus 200K diffractometer	3935 independent reflections
Radiation source: micro-focus sealed X-ray tube, SuperNova (Cu) X-ray Source	3924 reflections with *I* > 2σ(*I*)
Mirror monochromator	*R*_int_ = 0.024
ω scans	θ_max_ = 80.1°, θ_min_ = 3.4°
Absorption correction: multi-scan (CrysAlisPro; Rigaku OD, 2018)	*h* = −16→16
*T*_min_ = 0.507, *T*_max_ = 1.000	*k* = −19→18
20327 measured reflections	*l* = −8→11

### 5-Bromo-1,3-di-tert-butyl-2-[(trimethylsilyl)oxy]benzene (I) Refinement

**Table:**

Refinement on *F*^2^	Hydrogen site location: inferred from neighbouring sites
Least-squares matrix: full	H-atom parameters constrained
*R*[*F*^2^ > 2σ(*F*^2^)] = 0.031	*w* = 1/[σ^2^(*F*_o_^2^) + (0.0196*P*)^2^ + 3.634*P*] where *P* = (*F*_o_^2^ + 2*F*_c_^2^)/3
*wR*(*F*^2^) = 0.073	(Δ/σ)_max_ = 0.002
*S* = 1.10	Δρ_max_ = 0.66 e Å^−^^3^
3935 reflections	Δρ_min_ = −0.36 e Å^−^^3^
191 parameters	Extinction correction: SHELXL-2018/3 (Sheldrick 2015b), Fc^*^=kFc[1+0.001xFc^2^λ^3^/sin(2θ)]^-1/4^
0 restraints	Extinction coefficient: 0.00139 (10)
Primary atom site location: dual	

### 5-Bromo-1,3-di-tert-butyl-2-[(trimethylsilyl)oxy]benzene (I) Special details

**Table:**

Geometry. All esds (except the esd in the dihedral angle between two l.s. planes) are estimated using the full covariance matrix. The cell esds are taken into account individually in the estimation of esds in distances, angles and torsion angles; correlations between esds in cell parameters are only used when they are defined by crystal symmetry. An approximate (isotropic) treatment of cell esds is used for estimating esds involving l.s. planes.

### 5-Bromo-1,3-di-tert-butyl-2-[(trimethylsilyl)oxy]benzene (I) Fractional atomic coordinates and isotropic or equivalent isotropic displacement parameters (Å^2^)

**Table:**

	*x*	*y*	*z*	*U*_iso_*/*U*_eq_	
Br1	0.51566 (2)	0.21330 (2)	0.45554 (3)	0.01991 (9)	
Si1	0.81135 (4)	0.60159 (4)	0.69256 (6)	0.01462 (13)	
O1	0.80844 (10)	0.51233 (9)	0.58830 (15)	0.0137 (3)	
C1	0.74581 (14)	0.44182 (13)	0.5533 (2)	0.0122 (4)	
C2	0.67726 (14)	0.44492 (13)	0.4237 (2)	0.0124 (4)	
C3	0.60978 (15)	0.37556 (13)	0.3970 (2)	0.0139 (4)	
H3	0.562847	0.376592	0.314197	0.017*	
C4	0.61193 (15)	0.30530 (13)	0.4923 (2)	0.0143 (4)	
C5	0.68503 (15)	0.29845 (13)	0.6102 (2)	0.0136 (4)	
H5	0.686809	0.249093	0.670039	0.016*	
C6	0.75644 (15)	0.36495 (13)	0.6406 (2)	0.0128 (4)	
C7	0.67316 (15)	0.52100 (13)	0.3119 (2)	0.0141 (4)	
C8	0.60959 (17)	0.49792 (14)	0.1665 (2)	0.0183 (4)	
H8A	0.636634	0.446032	0.125038	0.027*	
H8B	0.612894	0.545090	0.097353	0.027*	
H8C	0.539601	0.488246	0.186543	0.027*	
C9	0.61968 (17)	0.59939 (14)	0.3782 (2)	0.0196 (4)	
H9A	0.548598	0.586074	0.385149	0.029*	
H9B	0.625605	0.649347	0.315599	0.029*	
H9C	0.651459	0.611686	0.475345	0.029*	
C10	0.78100 (17)	0.54395 (15)	0.2675 (2)	0.0200 (4)	
H10A	0.823327	0.561482	0.353977	0.030*	
H10B	0.776290	0.590827	0.196991	0.030*	
H10C	0.810620	0.493866	0.223875	0.030*	
C11	0.84163 (15)	0.35033 (13)	0.7659 (2)	0.0140 (4)	
C12	0.85418 (16)	0.25267 (14)	0.8049 (2)	0.0184 (4)	
H12A	0.793262	0.231898	0.845688	0.028*	
H12B	0.911767	0.245176	0.876394	0.028*	
H12C	0.865318	0.220270	0.716834	0.028*	
C13	0.94702 (15)	0.37909 (14)	0.7187 (2)	0.0183 (4)	
H13A	0.963697	0.345661	0.634250	0.027*	
H13B	0.998222	0.369667	0.799205	0.027*	
H13C	0.944785	0.439842	0.693388	0.027*	
C14	0.81303 (17)	0.39676 (14)	0.9074 (2)	0.0181 (4)	
H14A	0.800068	0.457243	0.886197	0.027*	
H14B	0.868505	0.391592	0.982939	0.027*	
H14C	0.752635	0.370455	0.941273	0.027*	
C15	0.69125 (17)	0.61128 (15)	0.7890 (2)	0.0223 (5)	
H15A	0.645076	0.650692	0.735070	0.033*	
H15B	0.707232	0.633097	0.887583	0.033*	
H15C	0.659611	0.555032	0.793856	0.033*	
C16	0.83126 (18)	0.69902 (14)	0.5749 (3)	0.0238 (5)	
H16A	0.888992	0.689285	0.518019	0.036*	
H16B	0.843886	0.749167	0.637185	0.036*	
H16C	0.771138	0.708748	0.509058	0.036*	
C17	0.92756 (18)	0.59976 (15)	0.8263 (3)	0.0247 (5)	
H17A	0.921155	0.553917	0.897176	0.037*	
H17B	0.934120	0.654670	0.877009	0.037*	
H17C	0.987189	0.589737	0.773474	0.037*	

### 5-Bromo-1,3-di-tert-butyl-2-[(trimethylsilyl)oxy]benzene (I) Atomic displacement parameters (Å^2^)

**Table:**

	*U*^11^	*U*^22^	*U*^33^	*U*^12^	*U*^13^	*U*^23^
Br1	0.01809 (13)	0.01465 (12)	0.02615 (14)	−0.00553 (8)	−0.00349 (8)	0.00120 (8)
Si1	0.0148 (3)	0.0125 (3)	0.0165 (3)	−0.00183 (19)	0.0007 (2)	−0.0017 (2)
O1	0.0135 (6)	0.0125 (6)	0.0153 (6)	−0.0022 (5)	0.0009 (5)	−0.0006 (5)
C1	0.0107 (8)	0.0117 (9)	0.0145 (9)	−0.0004 (7)	0.0029 (7)	−0.0023 (7)
C2	0.0120 (9)	0.0134 (9)	0.0121 (9)	0.0027 (7)	0.0024 (7)	−0.0002 (7)
C3	0.0128 (9)	0.0154 (9)	0.0132 (9)	0.0006 (7)	−0.0002 (7)	−0.0003 (7)
C4	0.0118 (9)	0.0130 (9)	0.0183 (10)	−0.0021 (7)	0.0021 (7)	−0.0029 (7)
C5	0.0140 (9)	0.0127 (9)	0.0142 (9)	0.0002 (7)	0.0018 (7)	0.0017 (7)
C6	0.0124 (9)	0.0146 (9)	0.0119 (9)	0.0015 (7)	0.0037 (7)	−0.0013 (7)
C7	0.0158 (9)	0.0129 (9)	0.0136 (9)	0.0005 (7)	0.0012 (7)	0.0015 (7)
C8	0.0228 (10)	0.0177 (10)	0.0142 (9)	0.0003 (8)	0.0000 (8)	0.0022 (8)
C9	0.0240 (11)	0.0149 (10)	0.0198 (10)	0.0054 (8)	−0.0002 (8)	−0.0007 (8)
C10	0.0210 (10)	0.0230 (11)	0.0166 (10)	−0.0039 (8)	0.0045 (8)	0.0029 (8)
C11	0.0139 (9)	0.0147 (9)	0.0131 (9)	0.0006 (7)	−0.0004 (7)	0.0006 (7)
C12	0.0175 (10)	0.0176 (10)	0.0197 (10)	0.0027 (8)	−0.0015 (8)	0.0037 (8)
C13	0.0132 (9)	0.0198 (10)	0.0215 (10)	0.0009 (8)	−0.0005 (8)	0.0020 (8)
C14	0.0204 (10)	0.0194 (10)	0.0142 (9)	0.0001 (8)	−0.0006 (8)	−0.0004 (8)
C15	0.0215 (11)	0.0246 (11)	0.0214 (11)	0.0014 (9)	0.0049 (8)	−0.0021 (9)
C16	0.0261 (11)	0.0157 (10)	0.0294 (12)	−0.0049 (9)	0.0021 (9)	0.0007 (9)
C17	0.0230 (11)	0.0209 (11)	0.0291 (12)	−0.0048 (9)	−0.0042 (9)	−0.0035 (9)

### 5-Bromo-1,3-di-tert-butyl-2-[(trimethylsilyl)oxy]benzene (I) Geometric parameters (Å, º)

**Table:**

Br1—C4	1.904 (2)	C10—H10A	0.9600
Si1—O1	1.6617 (15)	C10—H10B	0.9600
Si1—C15	1.865 (2)	C10—H10C	0.9600
Si1—C16	1.865 (2)	C11—C12	1.545 (3)
Si1—C17	1.866 (2)	C11—C13	1.542 (3)
O1—C1	1.379 (2)	C11—C14	1.537 (3)
C1—C2	1.419 (3)	C12—H12A	0.9600
C1—C6	1.420 (3)	C12—H12B	0.9600
C2—C3	1.392 (3)	C12—H12C	0.9600
C2—C7	1.543 (3)	C13—H13A	0.9600
C3—H3	0.9300	C13—H13B	0.9600
C3—C4	1.379 (3)	C13—H13C	0.9600
C4—C5	1.378 (3)	C14—H14A	0.9600
C5—H5	0.9300	C14—H14B	0.9600
C5—C6	1.397 (3)	C14—H14C	0.9600
C6—C11	1.541 (3)	C15—H15A	0.9600
C7—C8	1.540 (3)	C15—H15B	0.9600
C7—C9	1.538 (3)	C15—H15C	0.9600
C7—C10	1.540 (3)	C16—H16A	0.9600
C8—H8A	0.9600	C16—H16B	0.9600
C8—H8B	0.9600	C16—H16C	0.9600
C8—H8C	0.9600	C17—H17A	0.9600
C9—H9A	0.9600	C17—H17B	0.9600
C9—H9B	0.9600	C17—H17C	0.9600
C9—H9C	0.9600		
			
O1—Si1—C15	110.45 (9)	H10A—C10—H10B	109.5
O1—Si1—C16	109.57 (9)	H10A—C10—H10C	109.5
O1—Si1—C17	109.61 (9)	H10B—C10—H10C	109.5
C15—Si1—C16	111.39 (11)	C6—C11—C12	111.50 (16)
C15—Si1—C17	111.88 (11)	C6—C11—C13	111.48 (16)
C16—Si1—C17	103.75 (11)	C13—C11—C12	105.05 (16)
C1—O1—Si1	139.75 (13)	C14—C11—C6	109.51 (16)
O1—C1—C2	119.25 (17)	C14—C11—C12	106.75 (16)
O1—C1—C6	119.59 (17)	C14—C11—C13	112.41 (17)
C2—C1—C6	120.97 (18)	C11—C12—H12A	109.5
C1—C2—C7	123.59 (17)	C11—C12—H12B	109.5
C3—C2—C1	117.91 (18)	C11—C12—H12C	109.5
C3—C2—C7	118.50 (17)	H12A—C12—H12B	109.5
C2—C3—H3	119.7	H12A—C12—H12C	109.5
C4—C3—C2	120.58 (18)	H12B—C12—H12C	109.5
C4—C3—H3	119.7	C11—C13—H13A	109.5
C3—C4—Br1	119.37 (15)	C11—C13—H13B	109.5
C5—C4—Br1	119.17 (15)	C11—C13—H13C	109.5
C5—C4—C3	121.40 (18)	H13A—C13—H13B	109.5
C4—C5—H5	119.7	H13A—C13—H13C	109.5
C4—C5—C6	120.60 (18)	H13B—C13—H13C	109.5
C6—C5—H5	119.7	C11—C14—H14A	109.5
C1—C6—C11	124.24 (17)	C11—C14—H14B	109.5
C5—C6—C1	117.59 (18)	C11—C14—H14C	109.5
C5—C6—C11	118.17 (17)	H14A—C14—H14B	109.5
C8—C7—C2	111.93 (16)	H14A—C14—H14C	109.5
C8—C7—C10	105.93 (16)	H14B—C14—H14C	109.5
C9—C7—C2	109.21 (16)	Si1—C15—H15A	109.5
C9—C7—C8	106.33 (16)	Si1—C15—H15B	109.5
C9—C7—C10	112.26 (17)	Si1—C15—H15C	109.5
C10—C7—C2	111.09 (16)	H15A—C15—H15B	109.5
C7—C8—H8A	109.5	H15A—C15—H15C	109.5
C7—C8—H8B	109.5	H15B—C15—H15C	109.5
C7—C8—H8C	109.5	Si1—C16—H16A	109.5
H8A—C8—H8B	109.5	Si1—C16—H16B	109.5
H8A—C8—H8C	109.5	Si1—C16—H16C	109.5
H8B—C8—H8C	109.5	H16A—C16—H16B	109.5
C7—C9—H9A	109.5	H16A—C16—H16C	109.5
C7—C9—H9B	109.5	H16B—C16—H16C	109.5
C7—C9—H9C	109.5	Si1—C17—H17A	109.5
H9A—C9—H9B	109.5	Si1—C17—H17B	109.5
H9A—C9—H9C	109.5	Si1—C17—H17C	109.5
H9B—C9—H9C	109.5	H17A—C17—H17B	109.5
C7—C10—H10A	109.5	H17A—C17—H17C	109.5
C7—C10—H10B	109.5	H17B—C17—H17C	109.5
C7—C10—H10C	109.5		
			
Br1—C4—C5—C6	−179.69 (15)	C2—C3—C4—C5	−4.7 (3)
Si1—O1—C1—C2	95.3 (2)	C3—C2—C7—C8	−13.1 (2)
Si1—O1—C1—C6	−89.5 (2)	C3—C2—C7—C9	104.4 (2)
O1—C1—C2—C3	−175.38 (17)	C3—C2—C7—C10	−131.27 (19)
O1—C1—C2—C7	4.2 (3)	C3—C4—C5—C6	3.1 (3)
O1—C1—C6—C5	173.89 (17)	C4—C5—C6—C1	4.7 (3)
O1—C1—C6—C11	−6.3 (3)	C4—C5—C6—C11	−175.10 (17)
C1—C2—C3—C4	−1.6 (3)	C5—C6—C11—C12	18.1 (2)
C1—C2—C7—C8	167.30 (18)	C5—C6—C11—C13	135.20 (19)
C1—C2—C7—C9	−75.2 (2)	C5—C6—C11—C14	−99.8 (2)
C1—C2—C7—C10	49.1 (2)	C6—C1—C2—C3	9.5 (3)
C1—C6—C11—C12	−161.65 (18)	C6—C1—C2—C7	−170.85 (17)
C1—C6—C11—C13	−44.6 (3)	C7—C2—C3—C4	178.75 (18)
C1—C6—C11—C14	80.4 (2)	C15—Si1—O1—C1	−2.0 (2)
C2—C1—C6—C5	−11.0 (3)	C16—Si1—O1—C1	−125.1 (2)
C2—C1—C6—C11	168.74 (17)	C17—Si1—O1—C1	121.7 (2)
C2—C3—C4—Br1	178.09 (15)		

### 1,3-Di-tert-butyl-2-[(trimethylsilyl)oxy]benzene (II) Crystal data

**Table:**

C_17_H_30_OSi	*D*_x_ = 1.086 Mg m^−^^3^
*M**_r_* = 278.50	Cu *K*α radiation, λ = 1.54184 Å
Orthorhombic, *P**n**m**a*	Cell parameters from 12570 reflections
*a* = 14.47237 (14) Å	θ = 5.1–74.4°
*b* = 17.4657 (2) Å	µ = 1.13 mm^−^^1^
*c* = 6.73933 (7) Å	*T* = 100 K
*V* = 1703.50 (3) Å^3^	Needle, clear colourless
*Z* = 4	0.31 × 0.11 × 0.07 mm
*F*(000) = 616	

### 1,3-Di-tert-butyl-2-[(trimethylsilyl)oxy]benzene (II) Data collection

**Table:**

Rigaku Oxford Diffraction SuperNova, Dual, Cu at zero, Pilatus 200K diffractometer	1793 independent reflections
Radiation source: micro-focus sealed X-ray tube, SuperNova (Cu) X-ray Source	1663 reflections with *I* > 2σ(*I*)
Mirror monochromator	*R*_int_ = 0.046
ω scans	θ_max_ = 74.7°, θ_min_ = 5.1°
Absorption correction: multi-scan (CrysAlisPro; Rigaku OD, 2018)	*h* = −18→17
*T*_min_ = 0.773, *T*_max_ = 1.000	*k* = −21→20
17855 measured reflections	*l* = −8→8

### 1,3-Di-tert-butyl-2-[(trimethylsilyl)oxy]benzene (II) Refinement

**Table:**

Refinement on *F*^2^	Primary atom site location: dual
Least-squares matrix: full	Hydrogen site location: inferred from neighbouring sites
*R*[*F*^2^ > 2σ(*F*^2^)] = 0.033	H-atom parameters constrained
*wR*(*F*^2^) = 0.093	*w* = 1/[σ^2^(*F*_o_^2^) + (0.0463*P*)^2^ + 0.6892*P*] where *P* = (*F*_o_^2^ + 2*F*_c_^2^)/3
*S* = 1.06	(Δ/σ)_max_ < 0.001
1793 reflections	Δρ_max_ = 0.26 e Å^−^^3^
99 parameters	Δρ_min_ = −0.28 e Å^−^^3^
0 restraints	

### 1,3-Di-tert-butyl-2-[(trimethylsilyl)oxy]benzene (II) Special details

**Table:**

Geometry. All esds (except the esd in the dihedral angle between two l.s. planes) are estimated using the full covariance matrix. The cell esds are taken into account individually in the estimation of esds in distances, angles and torsion angles; correlations between esds in cell parameters are only used when they are defined by crystal symmetry. An approximate (isotropic) treatment of cell esds is used for estimating esds involving l.s. planes.

### 1,3-Di-tert-butyl-2-[(trimethylsilyl)oxy]benzene (II) Fractional atomic coordinates and isotropic or equivalent isotropic displacement parameters (Å^2^)

**Table:**

	*x*	*y*	*z*	*U*_iso_*/*U*_eq_	Occ. (<1)
Si1	0.44620 (3)	0.250000	0.02273 (7)	0.01982 (15)	
O1	0.55464 (8)	0.250000	0.10549 (17)	0.0194 (3)	
C1	0.59998 (11)	0.250000	0.2860 (2)	0.0181 (3)	
C2	0.62564 (8)	0.32076 (6)	0.37337 (17)	0.0195 (3)	
C3	0.66393 (8)	0.31815 (7)	0.56362 (18)	0.0228 (3)	
H3	0.679079	0.364859	0.628093	0.027*	
C4	0.68049 (12)	0.250000	0.6610 (3)	0.0244 (4)	
H4	0.702993	0.250001	0.793454	0.029*	
C7	0.61394 (8)	0.39980 (7)	0.27322 (18)	0.0219 (3)	
C8	0.67393 (10)	0.46122 (7)	0.3744 (2)	0.0309 (3)	
H8A	0.652833	0.468650	0.511229	0.046*	
H8B	0.668568	0.509554	0.301445	0.046*	
H8C	0.738594	0.444559	0.374947	0.046*	
C9	0.64519 (9)	0.39815 (7)	0.05482 (18)	0.0249 (3)	
H9A	0.710577	0.383808	0.047981	0.037*	
H9B	0.636715	0.448972	−0.004068	0.037*	
H9C	0.608163	0.360625	−0.018420	0.037*	
C10	0.51286 (9)	0.42590 (7)	0.29218 (19)	0.0268 (3)	
H10A	0.472354	0.388099	0.229055	0.040*	
H10B	0.505156	0.475597	0.226734	0.040*	
H10C	0.496631	0.430630	0.432822	0.040*	
C15	0.36580 (12)	0.250000	0.2393 (3)	0.0259 (4)	
H15A	0.306965	0.226595	0.200528	0.039*	0.5
H15B	0.354989	0.302788	0.282753	0.039*	0.5
H15C	0.393242	0.220617	0.348249	0.039*	0.5
C16	0.42870 (9)	0.16623 (8)	−0.1452 (2)	0.0282 (3)	
H16A	0.475983	0.166658	−0.249330	0.042*	
H16B	0.367304	0.169330	−0.206060	0.042*	
H16C	0.433624	0.118748	−0.068507	0.042*	

### 1,3-Di-tert-butyl-2-[(trimethylsilyl)oxy]benzene (II) Atomic displacement parameters (Å^2^)

**Table:**

	*U*^11^	*U*^22^	*U*^33^	*U*^12^	*U*^13^	*U*^23^
Si1	0.0205 (2)	0.0176 (3)	0.0213 (3)	0.000	−0.00179 (16)	0.000
O1	0.0212 (6)	0.0171 (6)	0.0198 (6)	0.000	−0.0020 (4)	0.000
C1	0.0178 (7)	0.0194 (8)	0.0170 (8)	0.000	0.0020 (6)	0.000
C2	0.0194 (5)	0.0178 (6)	0.0214 (6)	0.0005 (4)	0.0031 (4)	−0.0006 (5)
C3	0.0258 (6)	0.0213 (6)	0.0213 (6)	−0.0011 (5)	0.0027 (5)	−0.0037 (5)
C4	0.0277 (9)	0.0274 (9)	0.0182 (8)	0.000	0.0006 (7)	0.000
C7	0.0266 (6)	0.0154 (6)	0.0237 (6)	−0.0013 (5)	−0.0008 (5)	−0.0012 (5)
C8	0.0402 (8)	0.0188 (6)	0.0338 (7)	−0.0063 (5)	−0.0060 (6)	−0.0002 (5)
C9	0.0303 (6)	0.0189 (6)	0.0256 (6)	−0.0033 (5)	0.0024 (5)	0.0028 (5)
C10	0.0315 (7)	0.0197 (6)	0.0291 (7)	0.0050 (5)	0.0000 (5)	−0.0031 (5)
C15	0.0230 (8)	0.0266 (9)	0.0280 (9)	0.000	0.0007 (7)	0.000
C16	0.0297 (6)	0.0263 (7)	0.0286 (7)	0.0000 (5)	−0.0057 (5)	−0.0048 (5)

### 1,3-Di-tert-butyl-2-[(trimethylsilyl)oxy]benzene (II) Geometric parameters (Å, º)

**Table:**

Si1—O1	1.6655 (12)	C8—H8A	0.9800
Si1—C15	1.8664 (19)	C8—H8B	0.9800
Si1—C16	1.8671 (13)	C8—H8C	0.9800
Si1—C16^i^	1.8671 (13)	C9—H9A	0.9800
O1—C1	1.3821 (19)	C9—H9B	0.9800
C1—C2	1.4185 (14)	C9—H9C	0.9800
C1—C2^i^	1.4185 (14)	C10—H10A	0.9800
C2—C3	1.3976 (17)	C10—H10B	0.9800
C2—C7	1.5461 (16)	C10—H10C	0.9800
C3—H3	0.9500	C15—H15A	0.9800
C3—C4	1.3802 (15)	C15—H15B	0.9800
C4—H4	0.9500	C15—H15C	0.9800
C7—C8	1.5393 (17)	C16—H16A	0.9800
C7—C9	1.5400 (16)	C16—H16B	0.9800
C7—C10	1.5376 (17)	C16—H16C	0.9800
			
O1—Si1—C15	109.00 (7)	H8A—C8—H8B	109.5
O1—Si1—C16^i^	109.32 (5)	H8A—C8—H8C	109.5
O1—Si1—C16	109.32 (5)	H8B—C8—H8C	109.5
C15—Si1—C16^i^	112.92 (5)	C7—C9—H9A	109.5
C15—Si1—C16	112.92 (5)	C7—C9—H9B	109.5
C16—Si1—C16^i^	103.18 (9)	C7—C9—H9C	109.5
C1—O1—Si1	137.91 (10)	H9A—C9—H9B	109.5
O1—C1—C2	119.32 (7)	H9A—C9—H9C	109.5
O1—C1—C2^i^	119.32 (7)	H9B—C9—H9C	109.5
C2—C1—C2^i^	121.21 (15)	C7—C10—H10A	109.5
C1—C2—C7	124.62 (10)	C7—C10—H10B	109.5
C3—C2—C1	117.15 (11)	C7—C10—H10C	109.5
C3—C2—C7	118.24 (10)	H10A—C10—H10B	109.5
C2—C3—H3	118.9	H10A—C10—H10C	109.5
C4—C3—C2	122.23 (12)	H10B—C10—H10C	109.5
C4—C3—H3	118.9	Si1—C15—H15A	109.5
C3—C4—C3^i^	119.16 (16)	Si1—C15—H15B	109.5
C3—C4—H4	120.4	Si1—C15—H15C	109.5
C3^i^—C4—H4	120.4	H15A—C15—H15B	109.5
C8—C7—C2	111.54 (10)	H15A—C15—H15C	109.5
C8—C7—C9	105.71 (10)	H15B—C15—H15C	109.5
C9—C7—C2	111.61 (9)	Si1—C16—H16A	109.5
C10—C7—C2	109.43 (10)	Si1—C16—H16B	109.5
C10—C7—C8	107.06 (10)	Si1—C16—H16C	109.5
C10—C7—C9	111.37 (10)	H16A—C16—H16B	109.5
C7—C8—H8A	109.5	H16A—C16—H16C	109.5
C7—C8—H8B	109.5	H16B—C16—H16C	109.5
C7—C8—H8C	109.5		
			
Si1—O1—C1—C2	−92.19 (12)	C2^i^—C1—C2—C7	170.19 (9)
Si1—O1—C1—C2^i^	92.19 (12)	C2—C3—C4—C3^i^	3.9 (2)
O1—C1—C2—C3	174.50 (12)	C3—C2—C7—C8	17.63 (15)
O1—C1—C2—C7	−5.3 (2)	C3—C2—C7—C9	135.63 (11)
C1—C2—C3—C4	2.89 (19)	C3—C2—C7—C10	−100.63 (12)
C1—C2—C7—C8	−162.53 (12)	C7—C2—C3—C4	−177.26 (12)
C1—C2—C7—C9	−44.53 (16)	C15—Si1—O1—C1	0.000 (1)
C1—C2—C7—C10	79.20 (15)	C16^i^—Si1—O1—C1	123.87 (5)
C2^i^—C1—C2—C3	−10.0 (2)	C16—Si1—O1—C1	−123.87 (5)

Symmetry code: (i) *x*, −*y*+1/2, *z*.

### N-(2,6-Diisopropylphenyl)-1,1,1-trimethyl-N-(trimethylsilyl)silanamine (III) Crystal data

**Table:**

C_18_H_35_NSi_2_	*D*_x_ = 1.022 Mg m^−^^3^
*M**_r_* = 321.65	Mo *K*α radiation, λ = 0.71073 Å
Orthorhombic, *C**m**c**m*	Cell parameters from 4098 reflections
*a* = 12.199 (3) Å	θ = 2.4–27.5°
*b* = 12.091 (3) Å	µ = 0.17 mm^−^^1^
*c* = 14.177 (3) Å	*T* = 173 K
*V* = 2091.1 (8) Å^3^	Block, clear colourless
*Z* = 4	0.19 × 0.16 × 0.10 mm
*F*(000) = 712	

### N-(2,6-Diisopropylphenyl)-1,1,1-trimethyl-N-(trimethylsilyl)silanamine (III) Data collection

**Table:**

Bruker APEXII CCD diffractometer	1082 reflections with *I* > 2σ(*I*)
Graphite monochromator	*R*_int_ = 0.039
φ and ω scans	θ_max_ = 27.6°, θ_min_ = 2.4°
Absorption correction: ψ scan (SADABS; Bruker, 2014)	*h* = −15→15
*T*_min_ = 0.667, *T*_max_ = 0.746	*k* = −15→15
8996 measured reflections	*l* = −18→17
1317 independent reflections	

### N-(2,6-Diisopropylphenyl)-1,1,1-trimethyl-N-(trimethylsilyl)silanamine (III) Refinement

**Table:**

Refinement on *F*^2^	Primary atom site location: dual
Least-squares matrix: full	Hydrogen site location: inferred from neighbouring sites
*R*[*F*^2^ > 2σ(*F*^2^)] = 0.047	H-atom parameters constrained
*wR*(*F*^2^) = 0.126	*w* = 1/[σ^2^(*F*_o_^2^) + (0.0516*P*)^2^ + 2.3874*P*] where *P* = (*F*_o_^2^ + 2*F*_c_^2^)/3
*S* = 1.04	(Δ/σ)_max_ < 0.001
1317 reflections	Δρ_max_ = 0.36 e Å^−^^3^
64 parameters	Δρ_min_ = −0.26 e Å^−^^3^
54 restraints	

### N-(2,6-Diisopropylphenyl)-1,1,1-trimethyl-N-(trimethylsilyl)silanamine (III) Special details

**Table:**

Geometry. All esds (except the esd in the dihedral angle between two l.s. planes) are estimated using the full covariance matrix. The cell esds are taken into account individually in the estimation of esds in distances, angles and torsion angles; correlations between esds in cell parameters are only used when they are defined by crystal symmetry. An approximate (isotropic) treatment of cell esds is used for estimating esds involving l.s. planes.

### N-(2,6-Diisopropylphenyl)-1,1,1-trimethyl-N-(trimethylsilyl)silanamine (III) Fractional atomic coordinates and isotropic or equivalent isotropic displacement parameters (Å^2^)

**Table:**

	*x*	*y*	*z*	*U*_iso_*/*U*_eq_	Occ. (<1)
Si1	0.62813 (7)	0.91524 (6)	0.750000	0.0459 (3)	
N1	0.500000	0.8496 (2)	0.750000	0.0315 (6)	
C1	0.500000	0.7298 (2)	0.750000	0.0262 (6)	
C2	0.500000	0.67207 (17)	0.66401 (16)	0.0308 (5)	
C3	0.500000	0.55648 (18)	0.6663 (2)	0.0402 (6)	
H3	0.500000	0.516529	0.608650	0.048*	
C4	0.500000	0.4993 (3)	0.750000	0.0446 (9)	
H4	0.500000	0.420746	0.750000	0.053*	
C5	0.500000	0.7292 (2)	0.56902 (17)	0.0396 (6)	
H5	0.500000	0.810796	0.580380	0.047*	
C6	0.39760 (16)	0.70083 (19)	0.51164 (15)	0.0572 (5)	
H6A	0.395926	0.621120	0.499170	0.086*	
H6B	0.399211	0.741161	0.451700	0.086*	
H6C	0.332066	0.722035	0.547291	0.086*	
C7	0.7385 (2)	0.8087 (3)	0.750000	0.0656 (9)	
H7A	0.806954	0.841786	0.727270	0.098*	0.5
H7B	0.717550	0.747465	0.708470	0.098*	0.5
H7C	0.749174	0.780828	0.814260	0.098*	0.5
C8	0.6484 (3)	1.0030 (3)	0.8562 (2)	0.1024 (11)	
H8A	0.595639	1.064092	0.855311	0.154*	
H8B	0.723089	1.032743	0.856153	0.154*	
H8C	0.637079	0.958308	0.913029	0.154*	

### N-(2,6-Diisopropylphenyl)-1,1,1-trimethyl-N-(trimethylsilyl)silanamine (III) Atomic displacement parameters (Å^2^)

**Table:**

	*U*^11^	*U*^22^	*U*^33^	*U*^12^	*U*^13^	*U*^23^
Si1	0.0650 (5)	0.0344 (4)	0.0382 (4)	−0.0227 (3)	0.000	0.000
N1	0.0452 (14)	0.0211 (11)	0.0281 (13)	0.000	0.000	0.000
C1	0.0233 (13)	0.0231 (13)	0.0323 (15)	0.000	0.000	0.000
C2	0.0253 (9)	0.0293 (10)	0.0377 (12)	0.000	0.000	−0.0060 (9)
C3	0.0345 (11)	0.0283 (11)	0.0578 (16)	0.000	0.000	−0.0132 (10)
C4	0.0329 (16)	0.0222 (14)	0.079 (3)	0.000	0.000	0.000
C5	0.0492 (13)	0.0382 (12)	0.0313 (12)	0.000	0.000	−0.0087 (10)
C6	0.0526 (11)	0.0753 (14)	0.0437 (11)	0.0083 (10)	−0.0095 (9)	−0.0060 (10)
C7	0.0414 (15)	0.076 (2)	0.080 (2)	−0.0221 (15)	0.000	0.000
C8	0.117 (2)	0.0922 (19)	0.098 (2)	−0.0496 (19)	0.0103 (19)	−0.0548 (17)

### N-(2,6-Diisopropylphenyl)-1,1,1-trimethyl-N-(trimethylsilyl)silanamine (III) Geometric parameters (Å, º)

**Table:**

Si1—N1	1.7529 (13)	C1—C2^i^	1.405 (3)
Si1—C7	1.864 (4)	C2—C3	1.398 (3)
Si1—C8	1.858 (2)	C2—C5	1.513 (3)
Si1—C8^i^	1.858 (2)	C3—C4	1.373 (3)
N1—C1	1.448 (4)	C5—C6	1.530 (2)
C1—C2	1.405 (3)	C5—C6^ii^	1.530 (2)
			
N1—Si1—C7	109.36 (12)	C2—C1—N1	119.81 (13)
N1—Si1—C8	112.15 (10)	C2^i^—C1—C2	120.4 (3)
N1—Si1—C8^i^	112.15 (10)	C1—C2—C5	123.05 (19)
C8—Si1—C7	107.37 (13)	C3—C2—C1	118.5 (2)
C8^i^—Si1—C7	107.37 (13)	C3—C2—C5	118.5 (2)
C8—Si1—C8^i^	108.2 (2)	C4—C3—C2	121.6 (2)
Si1^iii^—N1—Si1	126.17 (15)	C3^i^—C4—C3	119.5 (3)
C1—N1—Si1^iii^	116.92 (7)	C2—C5—C6^ii^	111.78 (14)
C1—N1—Si1	116.92 (7)	C2—C5—C6	111.78 (14)
C2^i^—C1—N1	119.81 (13)	C6—C5—C6^ii^	109.5 (2)

Symmetry codes: (i) *x*, *y*, −*z*+3/2; (ii) −*x*+1, *y*, *z*; (iii) −*x*+1, *y*, −*z*+3/2.

## References

[bb1] Bruker (2014). *APEX2*, *SAINT* and *SADABS*. Bruker AXS Inc. Madison, Wisconsin, USA.

[bb2] Chung, M.-H., Yu, I. F., Liu, Y.-H., Lin, T.-S., Peng, S.-M. & Chiu, C. W. (2018). *Inorg. Chem.* **57**, 11732–11737. 10.1021/acs.inorgchem.8b01865 30179003

[bb3] Dolomanov, O. V., Bourhis, L. J., Gildea, R. J., Howard, J. A. K. & Puschmann, H. (2009). *J. Appl. Cryst.* **42**, 339–341.

[bb4] Goyal, M. & Singh, A. (1996). *Main Group Met. Chem.* **19**, 587–597.

[bb5] Groom, C. R., Bruno, I. J., Lightfoot, M. P. & Ward, S. C. (2016). *Acta Cryst.* B**72**, 171–179. 10.1107/S2052520616003954 PMC4822653 27048719

[bb6] Healy, M. D. & Barron, A. R. (1990). *J. Organomet. Chem.* **381**, 165–172.

[bb7] Kindra, D. R., Casely, I. J., Fieser, M. E., Ziller, J. W., Furche, F. & Evans, W. J. (2013). *J. Am. Chem. Soc.* **135**, 7777–7787. 10.1021/ja403133f 23621524

[bb8] Li, Y., Zhu, H., Andrada, D. M., Frenking, G. & Roesky, H. W. (2014). *Chem. Commun.* **50**, 4628–4630. 10.1039/c4cc00912f 24668031

[bb9] Lide, D. R. (2004). Editor. *CRC Handbook of Chemistry and Physics*, 85th ed, sect. 9.1. Boca Raton: CRC Press.

[bb10] Lucente-Schultz, R. M., Moore, V. C., Leonard, A. D., Price, B. K., Kosynkin, D. V., Lu, M., Partha, R., Conyers, J. L. & Tour, J. M. (2009). *J. Am. Chem. Soc.* **131**, 3934–3941. 10.1021/ja805721p 19243186

[bb11] Maaninen, A., Boeré, R. T., Chivers, T. & Parvez, M. (1999). *Z. Naturforsch. B*, **54**, 1170–1174.

[bb12] Marszaukowski, F. & Boeré, R. T. (2019). CSD Communication (refcode CCDC 1907965). CCDC, Cambridge, England. https://doi.org/10.5517/ccdc.csd.cc221d8t

[bb13] Nieves-Quinones, Y., Paniak, T. J., Lee, Y. E., Kim, S. M., Tcyrulnikov, S. & Kozlowski, M. C. (2019). *J. Am. Chem. Soc.* **141**, 10016–10032. 10.1021/jacs.9b03890 PMC6628261 31125210

[bb14] Otaki, M. & Goto, H. (2019). *Macromolecules*, **52**, 3199–3209.

[bb15] Pennington, D. A., Horton, P. N., Hursthouse, M. B., Bochmann, M. & Lancaster, S. J. (2005). *Polyhedron*, **24**, 151–156.

[bb16] Poverenov, E., Shimon, L. J. W. & Milstein, D. (2007). *Organometallics*, **26**, 2178–2182.

[bb17] Rigaku OD (2018). *CrysAlis PRO*. Rigaku Oxford Diffraction, Yarnton, England.

[bb18] Satoh, Y. & Shi, C. (1994). *Synthesis*, pp. 1146–1148.

[bb19] Sheldrick, G. M. (2015*a*). *Acta Cryst.* A**71**, 3–8.

[bb20] Sheldrick, G. M. (2015*b*). *Acta Cryst.* C**71**, 3–8.

[bb21] Siemeling, U., Neumann, B., Stammler, H.-G. & Kuhnert, O. (1999). *Polyhedron*, **18**, 1815–1819.

[bb22] Stalke, D., Keweloh, N., Klingebiel, U., Noltemeyer, M. & Sheldrick, G. M. (1987). *Z. Naturforsch. B*, **42**, 1237–1244.

[bb23] Wang, J., Pan, X., Liu, J., Zhao, L., Zhi, Y., Zhao, K. & Hu, L. (2018). *Org. Lett.* **20**, 5995–5998. 10.1021/acs.orglett.8b02127 30234990

[bb24] Westrip, S. P. (2010). *J. Appl. Cryst.* **43**, 920–925.

